# 

**Published:** 1916-04-15

**Authors:** 


### HOSPITAL RESULTS IN 1915.

### More Early Reports of the Voluntary Hospitals.

Children's Home Hospital, Strathblane.—The twelfth annual report records peaceful and uneventful progress, in which the outstanding feature is that the number of patients, who are Glasgow children, has increased in consequence of the military demands upon the accommodation of the city hospitals. As the average stay is rather lengthy, such of the children as are able attend a school in connection with the Home, which department is carried on under the regulations of the Scotch Education Department.

City of London Lying-in Hospital.—It is noticed that during the absence of Mr. H. Spencer Johnson on military service his administrative work is being carried on by the veteran hospital secretary, Mr. R. A. Owthwaite. The by-laws of the charity, happily brief, are printed in the annual report. In 1915, 1,011 women were delivered of 1,025 children—viz., 524 boys and 501 girls. Fourteen women had twins, forty-six children were stillborn, sixteen women and fifty-one infants died. The total income—£6,609—was rather less than in 1914, and the total expenditure £6,818 higher. There was an increase in the cost of provisions of 31 per cent. The premium for insurance against aircraft was £35.

Grimsby and District Hospital.—The annual report shows that the income and expenditure are each the largest in the hospital records, and that there was a surplus for 1915 of about £400. Mr. A. H. Leslie retires from the treasurership after service for a quarter of a century.

Royal Westminster Ophthalmic Hospital.—The work of the hospital was lighter in 1915 both in the wards and in the out-patient department. Of the out-patients many were soldiers and recruits temporarily below the standard of fitness prescribed by the authorities, and ten beds have been provided for military ophthalmic cases. The income was slightly less than in 1914, and the expenditure £250 higher. The secretary, Mr. J. H. Johnson, is serving in the Army Service Corps. The report is printed on thinner paper than last year, no doubt because of economical considerations, and the rules, medical tables, etc., are not given in this issue.

The Royal Infirmary, Edinburgh.—The year covered by the annual report and accounts is from October 1, 1914, to October 1, 1915, during which period 13,102 patients were treated in the wards; 5,771 of these were inhabitants of Edinburgh and Leith, and 6,581 came from country districts. Two spare wards and beds introduced in other wards have been devoted to the use of sailors and soldiers, a considerable number of men have been treated, including sick and injured sailors from the Grand Fleet and soldiers from France and the Dardanelles. During the year, 107 V.A.D. members received some brief instruction in the wards preparatory to entering as probationers in military hospitals. The total ordinary income was £46,474, being £10,141 more than in the preceding year; this very satisfactory state of affairs was mainly the result of a special appeal issued in the spring to which the response was widespread. Legacies and donations, counted as extraordinary receipts, amounted to £18,224. The total ordinary expenditure was £60,944, but the substantial gifts in kind received from the Colonies, etc., have not been valued for purposes of the accounts.

Westminster Hospital.—H.R.H. the Prince of Wales has been graciously pleased to accept the position of president of the hospital. The average number of beds occupied has risen from 174 to 179, and the average stay from twenty-five to thirty-one days. The total income was £24,289, and the expenditure £24,197; therefore, by using legacies amounting to £1,853, the hospital was just able to pay its way in 1915. The cost of provisions has risen by 22 per cent. 405 sick and wounded soldiers were treated. Out-patients fell from 15,713 to 13,262.

### PASSING EVENTS AND TOPICS.

### Increasing Cost of Patients.

The average cost of in-patients at the Bolton Infirmary for three years was stated at the annual meeting to be as follows: 1913-14, 4s. 5¼d.; 1914-15, 4s. 8¼d.; and 1915-16, 4s. 9¼d. An adverse balance on twelve months' working of £840 was reported.

### Limiting the Patients at Langho.

The Manchester Board of Guardians have decided to admit no new patients from outside institutions to their Langho Colony for sane epileptics. There is certified accommodation there for 250, but the inmates total 404, and an additional hospital building has to be used for them.

### The Islington Dispensary.

At the annual meeting, held on March 30, it was reported that the 1915 total of patients—6,734, making 45,380 attendances—was in excess of 1914. It is claimed that the bulk of the patients, particularly women and children, are not assisted by the National Insurance Act.

### Leicester Royal Infirmary.

The Board of Governors of the Leicester Royal Infirmary instructed the architect to proceed with the plans for building a new pathological department, at a cost of £4,500, at the end of the war. Sir Edward Wood has promised to give £1,000 towards the cost, and three other local gentlemen have promised nearly another thousand pounds.

### Watford District Hospital.

The annual meeting was held on April 3, when it was reported that but for a special appeal in 1914, with a record Hospital Saturday collection, the year 1915 would have been, financially, one of disaster. The Linen Guild and gifts from the girls of the Watford Grammar School provided all hospital linen requirements. During the year 1,057 patients were admitted.

### Mr. A. S. Brunt.

Mr. A. S. Brunt, on the secretarial staff of the Devonshire Hospital, Buxton, has been appointed secretary to the North Ormesby Hospital, Middlesbrough. Mr. Brunt began his career under Mr. Stevenson, general superintendent of the Devonshire Hospital, twelve years ago, and has had experience during that time at two other civil hospitals and a Poor-Law infirmary.

### Child Life and Welfare Exhibition.

The Association of Infant Welfare and Maternity Centres desires to make it known that the use of the name of the association in connection with the Child Life and Welfare Exhibition, which is to be held in London in June, is unauthorised. This matter had merely been under consideration by the Association, and it was decided that no part should be taken in the exhibition.

### The Royal Earlswood Institution.

Such institutions as the Earlswood Asylum are liable to be overlooked in war-time, and a recent appeal from this old-established institution deserves the consideration of the charitable. To hold the usual annual festival would be to court disaster, and the three or four thousand pounds generally forthcoming by its means have to be made up in other ways. There is a heavy debt to bankers and tradesmen at the present time which must give cause for anxiety to the executive.

### Hospital Saturday Fund.

Sir Thomas Vezey Strong presided at the annual general meeting on Wednesday evening, the 5th inst. The report of the Council stated that the income had steadily progressed; £36,809 had been received, as compared with £30,130 for the previous year. The total sum distributed in January last was £35,040, as against £28,008 in 1914, an increase of £7,032. The number of "Benefits" granted upon the application of the collectors was 46,472, some 6,000 less than in the preceding twelve months.

### Red Cross Day at a Departmental Store.

The funds of the Plymouth division of the British Red Cross Society have been augmented by £250 as a result of a Red Cross Day at a local emporium (Messrs. Spooner and Co.). A large number of ladies acted as saleswomen in the different departments, and the firm handed over to the Society £15 out of every £100 taken in cash, together with the entire proceeds of the provision and flower stalls, and the whole of the profit on the afternoon teas served.

### Cures for Cancer.

Sir George Beatson, speaking at the annual meeting of the Glasgow Royal Cancer Hospital, said that during the last eight years no fewer than twenty-six certain cures for cancer had been produced; they had had their day, and all of them had been found wanting. In the non-surgical treatment of cancer, for inoperable cases, the field at present was held by three agents—electricity, heat, and radium. Recently at the hospital, through the kindness of Mr. W. J. Chrystal, *x*-ray apparatus had been installed, and would be given a fair trial.

### Royal Hospital for Incurables, Putney.

Mr. R. J. Lesslie, a vice-president of the Royal Hospital, issues an appeal on behalf of those who find their way into the haven of refuge at Putney and the 700 out-pensioners who benefit by the charity. The yearly expenditure, averaging £35,000, is most difficult to maintain, as what may be described as "assured income" amounts only to £7,000. A luncheon at the Hall of the Grocers' Company on Friday, June 2, will take the place of the festival dinner of more settled times. It is hoped by the appeal on this occasion to wipe off the heavy debt of £9,000 to the bankers, and to lighten the task of the executive by providing further much-needed financial support.

### Royal Gifts at the Red Cross Sale.

The great sale at Christie's on behalf of the funds of the British Red Cross Society and the Order of St. John began on April 6 and will conclude on April 28. The first sale, which was held in April last year, realised nearly £40,000, but the present catalogue contains over 850 lots more than that of last year. The King's gift, which was offered on the first day, is a beautiful panel of Chinese embroidery. The Queen has given two hammered gold bracelets with diamond flower sprays, each being engraved with the inscription, "Given by Her Majesty Queen Mary to the British Red Cross Sale, 1916." The large Kaga-ware dish, with two mirrors and two scroll-shaped panels chosen by Queen Alexandra as a souvenir of King Edward VII.'s possessions, will be offered on April 18, and an Italian ebony writing-case, the gift of Princess Louise, will be sold on April 14.

### Gifts to Hospitals.

To the Nottingham Dispensary £150 has been bequeathed by Mrs. Jane Goodall, of Teignmouth.

The Tunbridge Wells General Hospital and the Sussex County Hospital have been left each £100 by Lord Abergavenny.

The West of Scotland Hospital for Limbless Soldiers and Sailors has received 1,000 guineas from Lord and Lady Newlands.

The Chelsea Hospital for Women has received a donation of £75 from the Court of Common Council of the City of London towards the Rebuilding Fund.

Miss Ann Margaret Grant, sister of the late Surgeon-General Grant, hon. surgeon to Queen Victoria, bequeathed £20,000 to the Northern Infirmary, Inverness.

The General Purposes Committee of the Birmingham City Council has decided to distribute £430, the amount of the Brinsley bequest, to Birmingham charities.

King Edward VII. Hospital, Windsor, and Addenbrooke's Hospital, Cambridge, each receive £100 under the will of the late Mr. J. E. Nixon, Senior Fellow of King's College, Cambridge.

Miss Agnes Thomson Stewart, of Mandau, Swords, county Dublin, left £4,300 in specific legacies to a number of charities, chiefly religious and medical, and the residue of her estate to hospitals and institutions of a like nature.

Mr. S. H. Renshaw, an old friend of Bury Infirmary, is endowing two beds in the institution, at a cost of £2,000, one to the memory of his late wife and the other to his son, a lieutenant of the Lancashire Fusiliers, who was killed at the Dardanelles.

### Personalia.

Mr. Charles H. Warren has been appointed secretary to the Royal Eye Hospital, Southwark, in succession to Mr. G. Norman Taylor, who is serving with the Forces. Mr. Warren, who is at present assistant secretary at the City of London Lying-in Hospital, was formerly secretary of the Metropolitan Provident Medical Association, a position which he held for twenty-five years. This institution was, however, closed through financial difficulties consequent on the operations of the National Insurance Act, complicated by the war. Mr. Warren will take up his duties at the Royal Eye Hospital on May 1. Mr. Conrad Thies has been acting as honorary secretary to the hospital since Mr. Taylor's departure.

### Obituary.

The death has occurred on active service of Major W. L. Hawksley, R.A.M.C., who was assistant medical officer of health at Liverpool until the outbreak of war. He was formerly house surgeon at the David Lewis Northern Hospital, Liverpool.

The Wellcome Historical Medical Museum, 54a Wigmore Street, Cavendish Square, W., will be closed for cleaning until April 30.

### Matrons' and Sisters' Appointments.

Bradford, City Hospital.—Miss Mary Graham has been appointed assistant matron. She was trained at Leeds Union Infirmary, and holds the certificate of the Central Midwives Board. Miss Graham, who until recently has been night sister at Bradford City Hospital, was previously ward sister at Monsall Fever Hospital and night sister at Stockport Borough Hospital.

Cardiganshire, Tregaron Hospital (King Edward VII. Welsh National Memorial Association).—Miss A. E. Jones has been appointed matron. Miss Jones, who is sister at Glan Ely Tuberculosis Hospital, Cardiff, was trained at Oldham Royal Infirmary.

Ham Green Sanatorium, Bristol.—Miss Florence Midgley has been appointed night superintendent, and Miss Marie Gregory ward sister. Miss Midgley was trained at the Kingston-upon-Hull Workhouse Infirmary, Anlaby Road, Hull, proceeding thence to Ruchill Hospital, Glasgow, as staff nurse, whilst she now holds the position of superintendent nurse at the Bedford Poor-Law Institution. Miss Gregory was trained at Meath Hospital, Dublin, and has since been nursing under the British Red Cross at Dublin Castle.

Hanwell Cottage Hospital.—Miss R. Fisher has been appointed matron. She has had varied experience, as after training at Croydon Infirmary she was staff nurse at the Royal Albert Docks branch of the Seamen's Hospital, night sister at West Norfolk and Lynn Hospital, and nurse-matron of Harlington Cottage Hospital. She has also nursed in Serbia under the British Red Cross.

Merthyr Tydfil, Pontsarn Hospital.—Mrs. N. Walters, trained at the Rutland and General Infirmary, Stamford, and since sister at Merthyr General Hospital, has been appointed matron of this hospital.

Newport, Mon., Cardigan House Surgical Hospital for Children.—Miss Elsie Edwards has been appointed matron. Miss Edwards, who was trained at Willesden Infirmary, has since been sister at Glan Ely Tuberculosis Hospital, Cardiff. The Cardigan House Surgical Hospital for Children is one of the institutions administered by the King Edward VII. Welsh National Memorial Association.

Paddington Green Children's Hospital.—Miss M. C. Tisdale has been appointed matron. Miss Tisdale is at present the assistant matron at St. Mary's Hospital, having previously been night superintendent there. She has had considerable experience in children's hospitals as sister at Queen Mary's Hospital, Carshalton, and ward sister and home sister at the Queen's Hospital for Children, Hackney Road, N.E. She was trained at King's College Hospital.

Perth Royal Infirmary.—Miss Clara Macgregor, who was trained at the Edinburgh Royal Infirmary, has been appointed theatre sister at this institution.

Queen Alexandra's Military Nursing Service for India.—Miss Lilian Mary Locke and Miss Theodora Maude Thornton have been appointed nursing sisters.

South London Hospital for Women, Clapham.—Miss Annie Jones Pearce has been appointed matron. She was trained at the Royal Free Hospital, becoming later theatre sister and surgical sister. She then proceeded to the City of London Hospital for Diseases of the Chest as night sister, and on the outbreak of war, being a member of the Royal Naval Nursing Service Reserve, she was called up and served at the Royal Naval Hospital, Plymouth. Later she worked in Serbia with the Anglo-Serbian Unit, returning to occupy the post of home sister at the City of London Hospital for Diseases of the Chest.

**NOTE.—Particulars of Appointments for insertion in this column will be welcomed.**

Rates of Subscription: Twelve months, 6/6; Six months, 4/-; Three months, 2/-, post free to any address in the United Kingdom. Abroad, Twelve months, 8/8; Six months, 5/-; Three months, 2/6, post free.—Address The Subscription Dept., "The Hospital." The Hospital Building, 28/29 Southampton Street, Strand, London, W.C.

